# Linking the Effect of Antibiotics on Partial-Nitritation Biofilters: Performance, Microbial Communities and Microbial Activities

**DOI:** 10.3389/fmicb.2018.00354

**Published:** 2018-02-26

**Authors:** Alejandro Gonzalez-Martinez, Alejandro Margareto, Alejandro Rodriguez-Sanchez, Chiara Pesciaroli, Silvia Diaz-Cruz, Damia Barcelo, Riku Vahala

**Affiliations:** ^1^Department of Built Environment, School of Engineering, Aalto University, Espoo, Finland; ^2^Department of Environmental Chemistry, Institute of Environmental Assessment and Water Research, Spanish Council for Scientific Research, Barcelona, Spain; ^3^Catalan Institute for Water Research, Scientific and Technological Park of the University of Girona, Girona, Spain; ^4^Institute of Water Research, University of Granada, Granada, Spain

**Keywords:** partial-nitritation, antibiotic resistance, metatranscriptomics, autotrophic nitrogen removal, microbial population, microbial activity

## Abstract

The emergence and spread of antibiotics resistance in wastewater treatment systems have been pointed as a major environmental health problem. Nevertheless, research about adaptation and antibiotics resistance gain in wastewater treatment systems subjected to antibiotics has not been successfully developed considering bioreactor performance, microbial community dynamics and microbial activity dynamics at the same time. To observe this in autotrophic nitrogen removal systems, a partial-nitritation biofilter was subjected to a continuous loading of antibiotics mix of azithromycin, norfloxacin, trimethoprim, and sulfamethoxazole. The effect of the antibiotics mix over the performance, bacterial communities and bacterial activity in the system was evaluated. The addition of antibiotics caused a drop of ammonium oxidation efficiency (from 50 to 5%) and of biomass concentration in the bioreactor, which was coupled to the loss of ammonium oxidizing bacteria *Nitrosomonas* in the bacterial community from 40 to 3%. Biomass in the partial nitritation biofilter experienced a sharp decrease of about 80% due to antibiotics loading, but the biomass adapted and experienced a growth by stabilization under antibiotics feeding. During the experiment several bacterial genera appeared, such as *Alcaligenes, Paracoccus*, and *Acidovorax*, clearly dominating the bacterial community with >20% relative abundance. The system reached around 30% ammonium oxidation efficiency after adaptation to antibiotics, but no effluent nitrite was found, suggesting that dominant antibiotics-resistant phylotypes could be involved in nitrification–denitrification metabolisms. The activity of ammonium oxidation measured as *amoA* and *hao* gene expression dropped a 98.25% and 99.21%, respectively, comparing the system before and after the addition of antibiotics. On the other hand, denitrifying activity increased as observed by higher expression of *nir* and *nos* genes (83.14% and 252.54%, respectively). In addition, heterotrophic nitrification *cyt c-551* was active only after the antibiotics addition. Resistance to the antibiotics was presumably given by *ermF, carA* and *msrA* for azithromycin, mutations of the *gyrA* and *grlB* for norfloxacin, and by *sul123* genes for sulfamethoxazole. Joined physicochemical and microbiological characterization of the system were used to investigate the effect of the antibiotics over the bioprocess. Despite the antibiotics resistance, activity of *Bacteria* decreased while the activity of *Archaea* and *Fungi* increased.

## Introduction

Several human and animal wastes, such as pharmaceutical industry effluents or livestock wastes, are treated through anaerobic digestion processes. In this way, anaerobic digestion offers an efficient treatment of these wastes due to low energy requirements, low sludge production and generation of methane as valuable subproduct, among others (Nasir et al., 2012). Concerning the threat of antibiotics resistance, the concentrations of antibiotics reported in pharmaceutical industry effluents and livestock wastes are in the range of 1–100 mg L^-1^ (Massé et al., 2014; Wang et al., 2015). In addition, the anaerobic digestion systems have shown poor antibiotics removal treatment in the range of 1–10 mg L^-1^ concentrations (Massé et al., 2014; Wang et al., 2015). In this sense, treatment systems handling effluent downstream anaerobic digestion processes should be able to withstand these antibiotics concentrations.

Anaerobic digestion supernatant is a residue obtained after anaerobic digestion process, which is characterized by high ammonium concentrations and low organic matter content. In the last 10 years, autotrophic nitrogen removal technologies have been developed for an efficient, cheap bioremediation of this waste (van der Star et al., 2007). These technologies rely on the unique metabolism of *“Candidatus Brocadiales”* bacteria, named as anaerobic ammonium oxidation, in which ammonium is oxidized using nitrite as terminal electron acceptor, yielding molecular nitrogen as a result (van Teeseling et al., 2016). In practical operation for the treatment of anaerobic digestion supernatant, the oxidation of half of the influent ammonium is required prior to the development of nitrogen removal through anaerobic ammonium oxidation. Thus, a partial-nitritation is a necessary previous step for the successful performance of autotrophic nitrogen removal technologies (van der Star et al., 2007). In order to develop and control separately the partial-nitritation and anaerobic ammonium oxidation, the partial-nitritation/anammox technology has been developed.

The purpose of the partial-nitritation process is to oxidize half of the influent ammonium for a subsequent treatment by *“Candidatus Brocadiales”* bacteria. Traditionally, partial-nitritation processes have been operated in suspended growth configuration. Nevertheless, attached growth partial-nitritation systems have been successfully operated, showing several advantages over suspended growth partial-nitritation processes such as lower hydraulic retention time (HRT) required (Rodriguez-Sanchez et al., 2016a,b). The ability of attached-growth partial-nitritation processes to operate under ciprofloxacin antibiotic pressure has been tested, showing that the system was impacted by the addition of the antimicrobial in terms of partial-nitritation performance and bacterial community structure (Gonzalez-Martinez et al., 2014b).

On the other hand, an emerging environmental and human health issue in the world today is the antibiotics resistant bacteria (Eramo et al., 2017; Zhu et al., 2017). Infectious bacteria resistant to antibiotics increase the costs of human health and increase the mortality of humans (Cao et al., 2016; Miller et al., 2016). Moreover, the bacterial evolution caused by antibiotics exposure could endanger the environmental health worldwide (Botts et al., 2017; Huang et al., 2017). Given that it has been reported that the introduction of antibiotics resistance to the environment is done by human and animal waste disposal, the wastewater treatment plants worldwide stand as a key element in the antibiotics resistance spread (Yu et al., 2016).

The bioprocess engineering science has usually used a monitoring approach for the study of bioreactors based on physicochemical determinations and evaluation of their microbial community structure (Gonzalez-Martinez et al., 2014b, 2015). Nevertheless, in terms of bioprocess functioning, the activity of the microorganisms is fundamental to the performance of biosystems, and thus the understanding of the metabolisms of the microbial communities in wastewater bioreactors (Rodriguez et al., 2015). To date, little work has been made in the investigation of metabolisms regarding the functioning of bioreactors (FitzGerald et al., 2015; Lawson et al., 2017). However, the effect of antibiotics over bioreactor functioning have never been attempted through metatranscriptomics approach.

For these reasons, the ability of an attached biofilm partial-nitritation process to handle high concentrations of an antibiotics mixture has been observed in terms of partial-nitritation performance, bacterial community structure dynamics and microbial community activity through metatranscriptomics. A synthetic wastewater emulating anaerobic digestion leachate from pharmaceutical wastewater treatment plant was used, containing different widely used antibiotics with different action mechanisms, such as macrolide azithromycin (AZT), quinolone norfloxacin(NOR) and sulfonamide trimethoprim (TMP)/sulfamethoxazole (SMZ), in high concentrations reported by several authors (Massé et al., 2014; Wang et al., 2015; Aydin et al., 2015; Meng F. et al., 2015; Meng L.W. et al., 2015). The results obtained showed the effect of the antibiotics addition over performance of the bioreactor and microbial activity.

## Materials and Methods

### Bioreactor Configuration, Start-up, and Operation

A lab-scale partial-nitritation biofilter was set-up for the experimentation in a similar approach based on the previous research (Gonzalez-Martinez et al., 2014a) (Supplementary Figure S1). The system was composed of a 5 L bioreactor filled in its whole volume with BioFlow9 carriers. The influent was introduced by the means of a peristaltic pump to achieve a HRT of 7 h. During the whole experiment, the temperature was controlled at 33 ± 1°C and the pH at 7.5 ± 0.2 with H_2_SO_4_ 0.1 M and NaOH 0.1 M for pH control. The aeration was constant, distributed equally through the bioreactor’s volume and maintained at 1.5 ± 0.3 mg-O_2_ L^-1^. The system was started-up using 1 L of activated sludge from the Los Vados WWTP (Granada, Spain) full-scale activated sludge bioreactor. For the start-up phase, synthetic wastewater simulating anaerobic digester supernatant was used following previous research on partial-nitritation biofilters (Gonzalez-Martinez et al., 2014b, 2016a). The composition of the wastewater is shown in **Table 1**. The start-up of the system was prolonged until a stable and efficient partial-nitritation performance under the optimal operational conditions to obtain 50% ammonium and 50% nitrite.

**Table 1:** Synthetic wastewater composition.

Chemical	Synthetic wastewater #1	Synthetic wastewater #2	Unit
(NH_4_)_2_SO_4_	2.35	2.35	g/L
NaHCO_3_	3.25	3.25	g/L
CaCl_2_	0.30	0.30	g/L
KH_2_PO_4_	0.07	0.07	g/L
MgSO_4_	0.02	0.02	g/L
FeSO_4_ 7H_2_O	0.009	0.009	g/L
H_2_SO_4_	0.005	0.005	g/L
Azithromycin	0	8	mg/L
Norfloxacin	0	2	mg/L
Sulfamethoxazole	0	9	mg/L
Trimethoprim	0	3	mg/L

The system was then operated for 30 days before to start the experiment under steady-state conditions. After this step, the experiment started from day 1 to day 60 days under steady-state conditions without antibiotics followed by 60 days (from day 60 to day 120) amended with antibiotics. For this purpose, the partial-nitritation bioreactor was continuously fed with wastewater #1 from day 1 to day 60 but from this day the influent wastewater composition was changed to wastewater #2 with a high antibiotics concentration containing AZT, NOR, SMZ, and TMP, which was maintained until the end of the experiment. Four antibiotics were continuously added to the influent to observe its effect on the partial-nitritation system. The synthetic wastewater composition with the antibiotics is shown in **Table 1**. The operation under antibiotics conditions was prolonged until the system reached steady-state conditions under the antibiotics loading, which occurred in a period of 2 months.

The operation of the bioreactor and the handling of biomass, influent and effluents were done following the Biosafety Level-2 from EU Directives 2000/54/EC on the protection of workers from risks related to exposure to biological agents at work.

### Determination of Nitrogenous Inorganic Compounds

The inorganic forms of nitrogen ammonium, nitrite, and nitrate were measured in the influent and effluent of the partial-nitritation biofilter on a daily basis by means of ionic chromatography.

### Determination of Biomass Concentration Attached to Carriers

The biomass attached to the BioFlow 9 carriers was evaluated daily. The method for its determination followed the procedure described previously for partial-nitritation biofilters (Gonzalez-Martinez et al., 2014b, 2016a).

### Determination of Antibiotics

Standards of AZT, NOR, SMZ, and TMP were supplied by Sigma-Aldrich (Steinheim, Germany). Deuterated standards AZT-d3, enrofloxacin-d5, sulfamethazine-d4, and TMP-d3, were supplied by Toronto Research Chemicals (Toronto, ON, Canada) and were of >99% purity. Methanol (MeOH), HPLC-grade water, and acetone LC-MS-grade solvents were purchased from Merck (Darmstadt, Germany) and acetonitrile (ACN) from Fischer Scientific (Loughborough, United Kingdom). High quality nitrogen (N_2_) and argon (Ar) were supplied by Abelló Linde (Barcelona, Spain).

Individual stock standard solutions of antibiotics were gravimetrically prepared in MeOH at 100 μg L^-1^. A 5 μg mL^-1^ internal standards (IS) solution and a stock standard solution of the mixture of all antibiotics at 5 and 1 μg mL^-1^, respectively, were prepared. Working mixture standards solutions were freshly prepared by appropriate dilution of the stock standard mixture solution in MeOH. Ten-point calibration curves were built in the range 10 ng L^-1^ to 1,500 ng L^-1^ and their pH was adjusted with HCOOH at 0.1% in volume before analysis. Calibration curves were run in every blanks and samples batch. All solutions were stored in the dark at -20°C and allowed to equilibrate at room temperature before use. Quantification of target antibiotics was performed by means of calibration curves obtained by linear regression analysis using the internal standardization on the basis of the best suited isotopically labeled compound for each analyte. The data were adjusted to linear least square regression curve with 1/x weighting index.

On-line solid phase extraction coupled to high performance liquid chromatography-tandem-mass spectrometry (on-line SPE-HPLC-MS/MS) (Margareto et al., unpublished) was used to determine the concentrations of AZT, NOR, TMP, and SMZ antibiotics in the influents and effluents of the partial-nitritation biofilter.

The influent and effluent water samples were conveniently diluted to fit into the calibration range, spiked with the IS mixture to reach a concentration of 500 ng L^-1^ and pH adjusted to 2.7. The automated on-line pre-concentration, purification and chromatographic separation of the analytes were performed using an on-line SPE–HPLC instrument Symbiosis^TM^ Pico (Spark Holland; Emmen, Netherlands). The elution of the retained antibiotics from the SPE cartridges (OASIS HLB) and the subsequent chromatographic separation was performed using a mobile phase consisted of HPLC-grade water (A) and ACN (B), both 0.1% in HCOOH. The separation was performed on a Purospher^®^ STAR RP-18 ec (125 mm × 2 mm, 5 μm particle size) LC-column from Merck (Darmstadt, Germany) with a guard column of the same material and setting a flow rate of 0.3 mL min^-1^. The following gradient was used (all steps linear): 0 min, 85% A; decreasing in 3 min to 20% A, kept constant for 7 min; 10 min 5% A;, kept constant for 2 min, and then returned to initial conditions in 3 min and, finally, 7 additional minutes to allow the column to equilibrate.

Detection was carried out in a 4000 QTRAP mass spectrometer (Applied Biosystems, Foster City, CA, United States) equipped with turbospray electrospray ionization (ESI) source. Data acquisition was performed in the positive ESI mode [ESI (+)] operated in selected reaction monitoring (SRM), allowing us to record two precursor ion-product ion mass transitions per compound. The most intense transition was used for quantification, and the other was used for confirmation, according to the identification and confirmation criteria for the analysis of drugs and other contaminants as defined by Commission Decision 2002/657/EC, implementing the Council Directive 96/23/EC. For data acquisition, peak area integration and quantification calculations the Analyst software v 1.5 (Sciex, Concord, ON, Canada) was used.

The experimental MS/MS parameters are listed in Supplementary Table S1. The method performance is summarized in Supplementary Table S2. Briefly; satisfactory average recovery rates ranging from 48.1 to 108.3% for influent and from 46.0 to 90.3% for effluent waters were achieved and expressed as the mean recovery values obtained at spike levels of 100 ng L^-1^, 250 ng L^-1^ and 500 ng L^-1^ with 3 replicates each. High sensitivity, expressed in terms of limits of detection (LOD) and quantification (LOQ) were reached, in the ranges 1.3–6.9 ng L^-1^ and 4.3–23.1 ng L^-1^, respectively, for influent and from 0.4 to 2.3 ng L^-1^ and from 1.5 to 7.8 ng L^-1^, respectively, for influent.

### Biomass Collection, DNA Extraction, and *iTag* High-Throughput Sequencing Procedure

For the purpose of characterization of bacterial community structure in the partial-nitritation biofilter, a total of 90 carriers (about 100 mL volume in total) was collected taking carriers distributed across the whole bioreactor’s volume. Biomass detachment from carriers was done according to previous procedures in partial nitritation biofilters (Gonzalez-Martinez et al., 2014b; Rodriguez-Sanchez et al., 2016a,b). Briefly, the carriers were sonicated for 3 min, then the detached biomass was submerged in saline solution (0.9% NaCl) and subjected to centrifugation at 3,500 rpm during 10 min at room temperature. The liquid supernatant was discarded and the collected biomass was kept at -20°C for subsequent DNA extraction.

The DNA extraction was done using the FastDNA SPIN Kit for Soil (MP Biomedicals, Solon, OH, United States) and the FastPrep apparatus following the instructions given by the manufacturer of the DNA extraction kit used and the procedure detailed in Gonzalez-Martinez et al. (2014a). The extracted DNA was then kept at -20°C and sent to Research and Testing Laboratory (Lubbock, TX, United States) for *iTag* high-throughput sequencing process.

The *iTag* high-throughput sequencing process was done using the Illumina MiSeq technology and the Illumina MiSeq Reagents Kit v3 at 2x300. The primers 28F-519R (5′-GAGTTTGATCNTGGCTCAG-3′ and 5′-GTNTTACNGCGGCKGCTG-3′, respectively), which have been used previously to determine the bacterial community structure of partial nitritation biofilters subjected to antibiotics (Gonzalez-Martinez et al., 2014b), were used for the amplification of the V1-V3 hypervariable regions of the 16S rRNA gene of *Bacteria*. The conditions of the PCR developed for the high-throughput sequencing were: 180 s at 94°C; 40 cycles of: 30 s at 94°C, 40 s at 60°C, 60 s at 72°C; 300 s at 72°C.

### Metatranscriptomic Analysis

The activities of bacterial communities in the bioreactor were determined by metatranscriptomic analyses at the beginning and the end of the operation under antibiotics pressure. A total volume of 100 mL of carriers samples were collected from the system and submerged into RNA Protect for the preservation of RNAs. The samples were kept at -80°C and sent to Research and Testing Laboratory.

The PowerMicrobiome RNA Isolation Kit (MOBIO, United States) was used for extraction of RNA and removal of genomic DNA from the samples following the instructions given by the manufacturer. The extraction process started with a lysis of cells using glass bead tubes and lysis solution. Then, a binding matrix captured all nucleic acids in the lysate, and DNA was removed by on-column DNase and wash solution, yielding only the RNA, which was preserved in RNase-free water for subsequent real time PCR.

The extracted RNA was then used for construction of libraries using the KAPA Stranded RNA-Seq Library Preparation Kit (KAPA Biosystems, United States) following the manufacturer’s instructions. The protocol started with fragmentation of RNA under by the means of heating and presence of Mg^+2^ with insert sizes ranging 200–300. Then, a conversion of first strand to cDNA using random primers was done, followed by a conversion of second strand to transform cDNA:RNA into double-stranded cDNA. dscDNA was then marked with dUTP and ligated to adapters. The adapter-ligated sequences library was then amplified by PCR. The library was sequenced using Illumina MiSeq technology and the Illumina MiSeq Reagents Kit v3 at 2x300. The raw sequences obtained are available in the SRA under the accession number SRP127026.

### *iTag* High-Throughput Sequencing Post-process

The analysis of the *iTag* high-throughput sequencing samples was done with the software mothur v1.34.4 (Schloss et al., 2009). First, paired-end reads were merged into contigs avoiding the generation of ambiguous bases in the overlap region. The contigs generated first passed a quality screening control to eliminate sequences with ambiguous bases and more than eight homopolymers. The remnant sequences were then aligned against the SiLVA SEED 123 release database, and those that failed to align properly were discarded for the analysis. Failure at alignment was regarded as: (i) failed to align at the position of the forward primer, and (ii) ended further than the 95% of the aligned sequences. The remaining sequences were then preclustered into a 2-bases threshold (Huse et al., 2010) and then checked for the presence of chimeric sequences using UCHIME v4.1 (Edgar et al., 2011), which were deleted from the analysis. After chimera deletion, the sequences were taxonomically affiliated and those that failed to classify within the domain *Bacteria* were eliminated.

To develop the bacterial ecology analysis of the *iTag* high-throughput sequencing samples, these were rarified and cut to form subsamples with 15778 sequences each. The sequences in each subsample were separately used to calculate a Phylip distance matrix between them, which was later utilized for the clustering of the sequences into OTUs within a 97% identity threshold. Representative sequences were then chosen for taxonomic classification of each of the OTUs using the SiLVA SEED 123 release database. The taxonomically affiliated OTUs were finally used to form a consensus taxonomy of OTUs within a cutoff of 80%.

### Ecological Analysis of the *iTag* Sequencing Subsamples

The *iTag* sequencing subsamples were subjected to an ecological analysis to determine their diversity coverage, α-diversity and β-diversity. For the diversity coverage, the Good’s coverage, the redundancy abundance-weighted coverage and the complexity curve of each subsample were calculated. The Good’s coverage was calculated using the species richness of each subsample in relation to their number of reads. The redundancy abundance-weighted coverage was calculated through NonPareil software taking a query set size of 1,000 sequences among the unique sequences within each subsample, allowing a minimum overlap of 50% and a 95% of identity between sequences (Rodriguez-R and Konstantinidis, 2014a,b). The complexity curves were calculated using aRarefactWin software. The Shannon–Wiener, Simpson, Chao1, Pielo’s evenness and Berger–Parker α-diversity indices were calculated using PAST software. The Morisita–Horn and symmetric β-diversity indices were calculated using the vegan 2.0 and vegetarian packages implemented in statistical software R.

### Post-process of Metatranscriptomic Data

The raw data obtained from the high-throughput sequencing of the cDNA were processed to yield information of the bacterial activity within the systems. The procedure mainly involved mapping against publicly available databases for the detection of rRNA, tRNA, and taxonomy and functional annotation of mRNA, as this is a common procedure for metatranscriptomics analyses (Aguiar-Pulido et al., 2016). Nevertheless, the use of closed reference databases restricts the results obtained to the scope of the information contained on these in terms of phylogenetic and functional affiliation of RNA.

For the metatranscriptomics pipeline, first, a quality trimming of sequences was done the paired-end sequences were merged into contigs using the software mothur v1.34.4 (Schloss et al., 2009) avoiding the appearance of ambiguous bases in the overlap region due to different nucleotide quality at the same position. Then, the contigs were trimmed to eliminate those with 1 or more ambiguous bases, 9 or more homopolymers, lower average quality score of 20 and length shorter than 200 bp.

Then, the remaining sequences were screened to eliminate those belonging to rRNA genes. This was done using BLAST software and the SiLVA Ref SSU and LSU databases, and sequences that found a match with an e-value < 10^-10^ were removed from the analysis. Then, an additional screening to eliminate other non-coding RNA sequences was done by BLAST search against non-coding RNA databases offered by RNACentral^1^. After the removal of non-coding RNA, the remaining sequences were thus considered mRNA.

The mRNA sequences were then BLASTed against several reference databases of genomes derived from RefSeq and NCBI for taxonomic affiliation. These databases were obtained from RefSeq^2^ and were used for the affiliation of the mRNA sequences to the taxonomic groups of *Archaea, Fungi, Protozoa, Virus*, and *Plasmids*. Also, an additional database of representative prokaryotic genomes derived from the NCBI^3^ was also used for classification of the mRNA in order to complete the taxonomic affiliation for the domain *Bacteria*. In all cases, matches were considered positive for bitscore >50 and e-value < 10^-5^ (Leimena et al., 2013; Mobberley et al., 2015).

The mRNA sequences that found a positive match with the RefSeq *Archaea* genomes database were then BLASTed against the corresponding RefSeq *Archaea* protein database for functional annotation. This was also true for the cases of *Fungi, Protozoa, Virus*, and *Plasmids*. The sequences that found a positive match with the NCBI representative prokaryotic genomes were BLASTed against the SwissProt database. The functional annotation was defined for e-values < 10^-5^, as suggested by previous authors (Leimena et al., 2013; Sayadi et al., 2016).

### Multivariate Redundancy Analyses

The operational parameters of the partial-nitritation biofilter (effluent ammonium, nitrite and nitrate concentrations; total nitrogen removal; biomass concentration; removal efficiencies of antibiotics AZT, NOR, TMP, and SMZ) were linked to the bacterial community structure of the partial-nitritation biofilter and the activity of its microbial communities through two multivariate redundancy analyses. These were done using the software CANOCO 4.5 for Windows and calculated through 499 unconstrained Monte-Carlo simulation under a full permutation model.

## Results and Discussion

### Partial-Nitritation Performance of the Partial-Nitritation Biofilter

**FIGURE 1:**
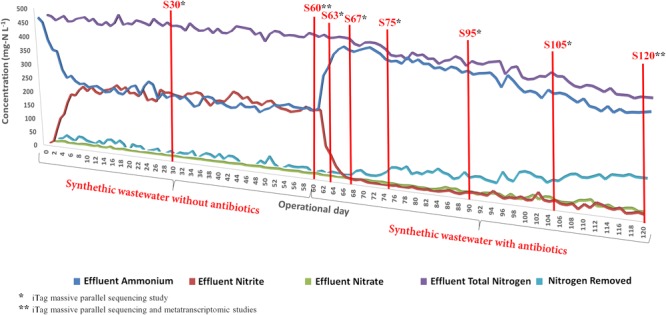
Performance of the partial-nitritation biofilter during the experiment.

Once the system reached steady-state conditions, the partial nitritation performance was nearly ideal, with around 50% of ammonium oxidized to nitrite and negligible nitrate concentrations during the 60 days under the synthetic wastewater conditions. The performance of the partial-nitritation biofilter under these conditions was similar to previous experiments (Gonzalez-Martinez et al., 2014b). Nevertheless, the addition of the antibiotics mix to the influent severely impacted the ammonium oxidation capacity (**Figure 1**). The partial-nitritation biofilter showed a reduction in ammonium oxidation efficiency from around 50% to around 5% after 6 days of the addition of the antibiotics. The system reached higher ammonium oxidation efficiencies over time, finishing in a steady-state value of around 30%. In this sense, the addition of antibiotics in high concentrations caused an irreversible loss of performance of the partial-nitritation biofilter. This has also been observed at low concentrations of ciprofloxacin antibiotics (Gonzalez-Martinez et al., 2014a). A sharp decrease in the effluent nitrite was observed coupled to the loss in ammonium oxidation. However, after 80 days of operation (20 days with antibiotics), a higher ammonium oxidation performance was observed. In this way, the increase in ammonium oxidation was not correlated with an increase in effluent nitrite concentration, but with an increase in nitrogen removal from the system. In this sense, it is possible that the addition of antibiotics triggered denitrification metabolisms from nitrite when the antibiotics or other organic matter within the biofilm were used as organic matter. This has also been observed in partial nitritation biofilters subjected to amino acids and antibiotics loading (Gonzalez-Martinez et al., 2014b, 2016a,b).

**FIGURE 2:**
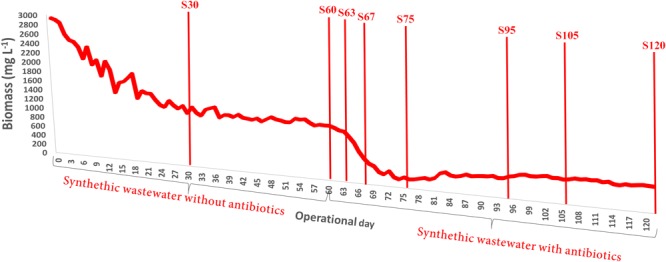
Evolution of the attached biofilm concentration in the partial-nitritation biofilter during the experiment.

The addition of the antibiotics mix caused an effect on the attached biofilm in the partial-nitritation biofilter, with a sharp decrease after the addition (**Figure 2**). With ongoing operation under the antibiotics mix the biomass slowly increased, but never could reach the values before the antibiotics addition. In this sense, the performance of the system was also influenced by the loss of biomass. This result resembles the one obtained for biomass growth in partial nitritation biofilters under different ciprofloxacin concentrations, with the exception that the system reached higher biomass concentrations under stable operations with antibiotic loading (Gonzalez-Martinez et al., 2014b). The differences may be driven by the differences in antibiotics concentrations between the two experiments, which were about 10^6^-fold, and the presence of a mix of antibiotics against only one compound. In this sense, the higher concentrations and the presence of several antibiotic compounds could exert more pressure over the partial nitritation biomass.

### Removal of Antibiotics

A net removal of the antibiotics AZT, NOR, TMP, and SMZ was observed during the experiment time (**Table 2**). SMZ was the antibiotic with lower removal efficiencies, showing a mean of 7.24 ± 4.90% during the operation under antibiotics addition. The higher mean was for AZT with 44.94 ± 14.55%, while for NOR and TMP were of 32.74 ± 9.63% and 32.59 ± 10.31%, respectively. Interestingly, the removal of AZT decreased significantly from day 90 to day 120, which might be explained by exhaustion of the adsorption capacity of the biofilm for AZT antibiotic, since reports showed that only around 10% AZT in WWTPs could be sorbed to biomass (Ivanová et al., 2017). Norfloxacin, SMZ, and trimethoprim were found to be removed by activated sludge process rather than becoming attached to biofilms (Yan et al., 2014a,b), and therefore their removal in this experiment could be attributed to degradation. The removal efficiencies at operational day 75 were the lowest for NOR, TMP, and SMZ. This might be caused by a turnover of microbial species within the bioreactor at that time. Also, the sorption of AZT, NOR, and SMZ could cause a net removal during the operation of the partial-nitritation biofilter.

**Table 2:** Antibiotics concentration (mg L^-1^) in the influent used in the experimental bioreactor and percentage of antibiotics removal during the experiment.

			Azithromycin	Norfloxacin	Sulfamethoxazole	Trimethoprim
Influent (mg L^-1^)			8.0	2.0	9.0	3.0
		
Removal (%)	Sample name	Operational time (days)	Azithromycin	Norfloxacin	Sulfamethoxazole	Trimethoprim
	S30^∗^	30	–	–	–	–
	S60^∗∗^	60	–	–	–	–
	S63	63	49.66	26.49	2.30	35.23
	S67	67	44.23	35.60	10.89	36.52
	S75	75	51.22	17.99	0.00	17.30
	S90	90	60.02	43.63	11.37	37.69
	S105	105	47.24	41.56	8.17	23.39
	S120	120	17.30	31.15	10.70	45.42

*^∗^Without antibiotics in the influent. ^∗∗^First day with addition of antibiotics in the influent.*

### Ecological Analysis of the *iTag* High-Throughput Sequencing Subsamples

The coverage of the *iTag* sequencing subsamples was sufficient to capture the bacterial diversity of the partial-nitritation biofilter. In this sense, the Good’s coverage index showed more than 97.4% coverage, while the redundancy abundance-weighted coverage had a minimum of 92.5% (Supplementary Table S3). Along with results obtained from complexity curves (Supplementary Figure S2), the coverage of the high-throughput sequencing subsamples seemed to be successful. Overall, the partial-nitritation biofilter without antibiotics addition had higher species richness than the system under the antibiotics loading, which found its lower diversity at the end of the antibiotics experiment. In this sense, the complexity curves showed a loss in diversity as the system was operated with antibiotics influent.

The Chao-1 index, which is related closely to species richness, showed its higher value at operational day 60 (S60), then decreased constantly until day 120 and had a slight increase by the end of the experiment (Supplementary Table S4), as showed by the complexity curves. Pielou’s evenness and Simpson indices values indicated that the diversity of the system decreased with operation time, showing that the antibiotics addition exerted an efficient selection that allowed few bacterial phylotypes to thrive under the antibiotics addition. In this sense, the evenness was lowest during operational days 75 (S75) and 90 (S90). The patterns regarding evenness of the bacterial communities in the partial-nitritation biofilter during the antibiotics experiment was also shown by the Berger–Parker index. Thus, the Shannon–Wiener index showed an increase from operational days 30 to 63 due to increase in bacterial diversity and evenness, and a continuous decrease as the antibiotics were added due to loss in bacterial diversity and evenness. Therefore, the values of α-diversity suggested that the adaptation of the system to the antibiotics mix influenced its bacterial community structure in terms of species diversity and evenness. Nevertheless, the lack of replicates in biological samples sets a limitation over the data collected regarding some of these indices, such as Chao-1.

The Morisita–Horn and the symmetric indices for the pair of *iTag* sequencing subsamples of interest are represented in Supplementary Figure S3. It could be found that the dominant bacterial phylotypes at operational days 30 (S30) and 60 (S60) were very different, as shown by high Morisita–Horn index value, while their rare phylotypes were of low similarity due to low symmetric index values. The dominant genera changed after the addition of the antibiotics and never recovered during the operation time, while the rare species persisted in the system. The dominant phylotypes changed drastically from day 60 (S60) to day 63 (S63), followed by a mild change from day 63 (S63) to day 67 (S67), and a period of acclimation from day 67 (S67) up to day 90 (S90). Nevertheless, from day 90 (S90) to day 105 (S105) a substantial change was observed, and then another stabilization was reached at day 120 (S120).

### Bacterial Community Dynamics in the Partial-Nitritation Biofilter

**FIGURE 3:**
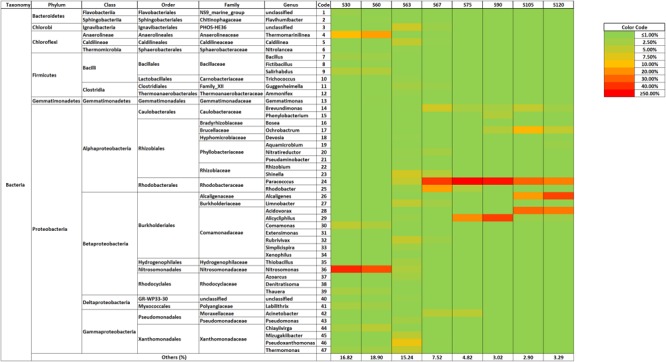
Bacterial community structure of the high-throughput sequencing subsamples.

The bacterial community dynamics showed that the addition of the antibiotics mix caused a deep change in the bacterial community structure of > 1% phylotypes in the partial-nitritation biofilter (**Figure 3**).

The first two pyrosequencing samples (days 30 and 60) were taken when the physico-chemical performance was stable in the partial nitritation bioreactor. In this way, it could be proven that the microbial population changes were caused by the antibiotics addition. Thus, at operational days 30 and 60, when the biofilter was fed with no-antibiotics influent, the bacterial community structure was clearly dominated by ammonium oxidizing *Nitrosomonas* genus, also with proliferation of strictly anaerobic *Chloroflexi*-belonging *Thermomarinilinea* (Nunora et al., 2013), which could develop anaerobic cell material degradation by utilization of *N*-acetylglucosamine within the biofilm as other members of its phylum (Gonzalez-Martinez et al., 2016a,b); heterotrophic nitrifier-aerobic denitrifier *Comamonas*, which has been found previously in partial-nitritation biofilters (Gonzalez-Martinez et al., 2016a); *Salirhabdus*, another aerobic denitrifier (Albuquerque et al., 2017); and *Chiayiivirga*, an aerobic, heterotrophic bacteria (Hsu et al., 2013). The bacterial community structure could be related to the performance of the bioreactor, with dominant *Nitrosomonas* linked to ammonium oxidation as observed in previous experimentations on partial-nitritation biofilters (Gonzalez-Martinez et al., 2014a,b, 2015, 2016a; Rodriguez-Sanchez et al., 2016a,b).

The Morisita–Horn index analysis showed that antibiotics caused significant changes in the bacterial community structure of the partial-nitritation biofilter. Accordingly, the analysis of bacterial dynamics showed that the addition of the antibiotics mix to the influent of the partial-nitritation biofilter caused a great decrease of *Nitrosomonas* relative abundance (from around 35–40% to 3%). The impact of the antibiotics caused loss of biomass and the proliferation of *Pseudoxanthomonas, Shinella, Rubrivivax, Thermomonas*, or *Paracoccus*, among others. After 7 days of antibiotics addition *Paracoccus* dominated the system at the same level of abundance as *Nitrosomonas* did with no antibiotics. *Rhodobacter* and *Brevundimonas* were also of importance at this operational day. *Paracoccus* was also the clearly dominant genus at operational day 75 (S75) followed by *Alicycliphilus*, with *Acinetobacter, Rhodobacter* and *Brevundimonas* being important but at much lower relative abundance. *Paracoccus* and *Alicycliphilus* still dominated the system by operational day 90 (S90), in which also important populations of *Ochrobactrum, Phenylobacterium*, and *Brevundimonas* appeared. By operational day 105 (E45) the genus *Alicycliphilus* had a very low presence in comparison with its abundance at operational day 90 (S90). *Paracoccus* still dominated, but its abundance was evenly matched with *Acidovorax*, with *Alcaligenes* and *Ochrobactrum* falling not far behind. By the last operational day 120 (S120) the domination belonged to *Alcaligenes*, followed by *Acidovorax* and *Paracoccus* which were also significantly and equally abundant. Interestingly, genera *Paracoccus, Rhodobacter, Brevundimonas, Alicycliphilus, Acinetobacter, Acidovorax* and *Alcaligenes* have been reported for denitrification metabolisms (Oosterkamp et al., 2013; Lu et al., 2014; Ji et al., 2015; Chen et al., 2016a,b; Qu et al., 2016; Yang et al., 2016; Tsubouchi et al., 2017). The presence of *Alcaligenes, Paracoccus*, and *Acidovorax* after the addition of an antibiotics mix of AZT, NOR, SMZ, and TMP was also found in a CANON bioreactor (Rodriguez-Sanchez et al., 2017). In this sense, these three genera are able to develop multi-antibiotics resistance and their importance in the proliferation and spread of antibiotics resistance genes in wastewater treatment systems should be explored.

### Activity in the Partial-Nitritation Biofilter in Absence and Presence of the Antibiotics Mix

#### Overview of Microorganisms Groups *Archaea, Bacteria, Fungi*, and *Protozoa*

The mRNA profile of the partial-nitritation biofilter before the addition of the antibiotics mix and at the end of the experiment were monitored using a metatranscriptomic approach. In this sense, results suggested notable differences in the activity of microorganisms within the biofilter (**Table 3**). As such, before the antibiotics addition, the majority of the mRNA (63.59%) was affiliated to the *Bacteria* domain followed by *Protozoa* group (31.45%), with *Archaea* and *Fungi* having a very low relative abundance (0.69% and 0.63%, respectively) in the general activity within the system. On the other hand, operation under the antibiotics conditions resulted in lower activity of *Bacteria* (54.32%) and *Protozoa* (25.91%) and a 10-fold increase in the activities of *Archaea* (8.26%) and *Fungi* (8.35%) with respect to the no-antibiotics scenario. Therefore, the mRNA profile showed that the addition of antibiotics increased the activities of *Archaea* and *Fungi* in the partial-nitritation biofilter. This could be caused by the lower susceptibility of archaeal and fungal phylotypes to the antimicrobials AZT, NOR, TMP, and SMZ used in the experiment.

**Table 3:** Taxonomic classification of mRNA found in the metatranscriptomics samples.

	Taxonomy			
	Before antibiotics (Day 60)		After antibiotics (day 120)	
	Reads	Identified Percent	Reads	Identified Percent
Identified mRNA	38144	100.00	13299	100.00
Prokaryotes	24255	63.59	7224	54.32
Archaea	265	0.69	1099	8.26
Fungi	242	0.63	1110	8.35
Virus	122	0.32	12	0.09
Plasmid	1264	3.31	408	3.07
Protozoa	11996	31.45	3446	25.91

#### The Activity of *Bacteria* Domain

**FIGURE 4:**
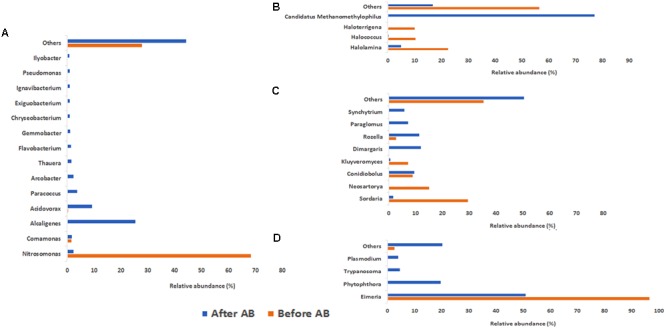
Contribution to mRNA activity at genus level of *Bacteria* **(A)**, *Archaea* **(B)**, *Fungi* **(C)**, and *Protozoa* **(D)** as determined by metatranscriptomic analysis. Before AB: before the antibiotics addition; After AB: after the antibiotics addition.

**FIGURE 5:**
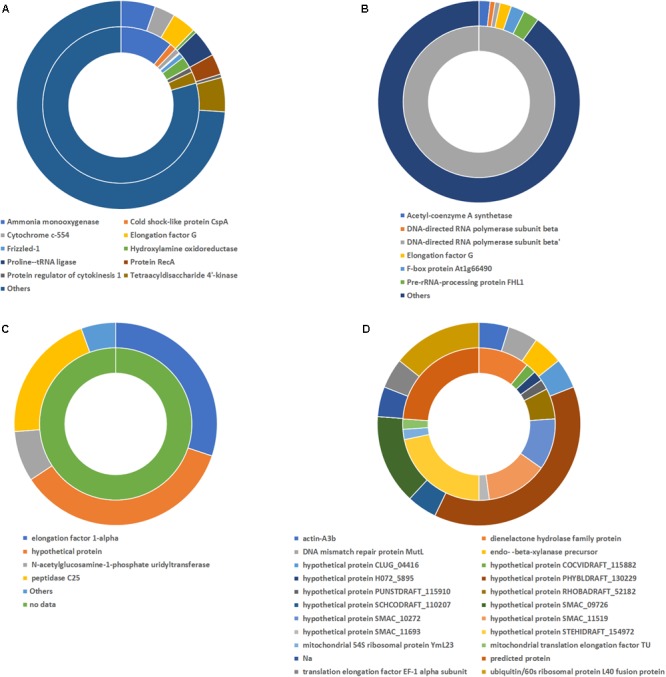
Activity profile of genera *Nitrosomonas* **(A)**, *Alcaligenes* **(B)**, *Candidatus Methanomethylophilus* **(C)**, and *Sordaria* **(D)** as determined by metatranscriptomic analysis. The inner circle represents the activity before the antibiotics addition and the outer circle that after the antibiotics addition.

In addition, changes in the global activity of the different microorganisms groups were observed. In the case of *Bacteria* at the no-antibiotics scenario, the dominant genera in terms of mRNA presence was *Nitrosomonas* (68.46%) (**Figure 4A**), which is related to the ammonium oxidation metabolism. This suggested the crucial role of *Nitrosomonas* in the ammonium oxidation in partial-nitritation biofilters, since ammonia monooxygenase activity (7.90% of total mRNA identified in the no-antibiotic scenario) was related to high partial-nitritation performance (about 50% ammonium-nitrite) and high relative abundance of *Nitrosomonas* (34.17%). Nevertheless, after the operation under the antibiotics mix, ammonia monooxygenase and hydroxylamine oxidoreductase had a 0.16% and 0.016%, respectively, along with the loss of *Nitrosomonas* relative abundance (0.00%) and partial-nitritation performance (around 70% ammonium-2.7% nitrite). *Nitrosomonas* did not change its dominant activity profile with respect to the antibiotics addition, being ammonia monooxygenase at both scenarios (10.90% and 5.32%, respectively), while a certain decrease in the ammonium oxidation was observed after the addition of the antibiotics (**Figure 5A**).

After the addition of the antibiotics, the most expressed proteins corresponded to *Alcaligenes* (25.56%), *Acidovorax* (9.34%), and *Paracoccus* (3.89%). We observed no other transcripts from *Alcaligenes* besides the DNA-directed RNA polymerase subunit β’ prior to the addition of antibiotics. However, after the operation under the antibiotics addition, the activity of this genus had a high diversity with predomination of elongation factor G (1.87%) or acetyl-coenzyme A synthase (1.82%), among others, while DNA-directed RNA polymerase subunit β and DNA-directed RNA polymerase subunit β’ were still found at high relative abundances (0.83% and 0.83%, respectively) (**Figure 5B**). The presence of acetyl-coenzyme A synthase indicated the heterotrophic metabolism of this genus.

#### The Activity of *Archaea* Domain

With respect to the domain *Archaea* under no antibiotics, the most active genera were *Halolamina* (22.64%), *Halococcus* (10.57%), *Haloterrigena* (10.19%), *Halalkalicoccus* (5.66%), and *Methanosarcina* (4.91%), among others. On the other hand, the domination under antibiotics operation belonged to *Candidatus Methanomethylophilus* (77.25%), followed by *Methanobacterium* (6.37%), *Halolamina* (5.10%), and *Methanosarcina* (1.46%) (**Figure 4B**). Interestingly, the clear dominance of *Methanomethylophilus* at the antibiotics scenario showed that this microorganism had a wide diversity of mRNA, with predominance of the elongation factor 1-alpha (30.11%), the peptidase C25 (20.69%) and the *N*-acetylglucosamine-1-phosphate uridyltransferase (8.05%) (**Figure 5C**). The elongation factor 1-alpha has been proposed as an omnipresent mechanism for quality control, elongation and termination of protein synthesis in *Archaea* (Saito et al., 2010). Also, peptidase C25 was found to be released by marine archaeal phylotypes for the degradation of proteins in marine sediments (Lloyd, 2013), and its high presence in the activity profile of *Methanomethylophilus* may signify a heterotrophic metabolism. *N*-acetylglucosamine-1-phosphate uridyltransferase, on the other hand, is related to cell wall material formation (Zhang et al., 2009).

#### The Activity of *Fungi* Microorganisms

Within the *Fungi* members, the activity profile changed from a domination of *Sordaria* (29.75%), *Neosartorya* (15.29%), *Conidiobolus* (9.09%), and *Kluyveromyces* (7.44%) to a composition mainly formed by *Dimargaris* (12.07%), *Rozella* (11.53%), *Conidiobolus* (9.64%), and *Paraglomus* (7.39%) (**Figure 4C**). The activity of *Sordaria* mainly changed from chlorocatechol degradation by expression of dienelactone hydrolase (10.87%) to glycan degradation expressed by high relative abundance of endo β xylanase precursor (4.76%) (**Figure 5D**).

#### The Activity of *Protozoa* Microorganisms

At the no-antibiotics scenario, the protozoan mRNA was almost exclusively affiliated to *Eimeria* (96.70%). Nevertheless, after operation under antibiotics, its affiliated mRNA dropped in half (51.16%), yielding an ecological niche for other protozoa, such as *Phytophthora* (19.76%), *Trypanosoma* (4.64%), and *Plasmodium* (4.06%), to grow (**Figure 4D**).

#### The Activity Concerning the Nitrogen Cycle

**FIGURE 6:**
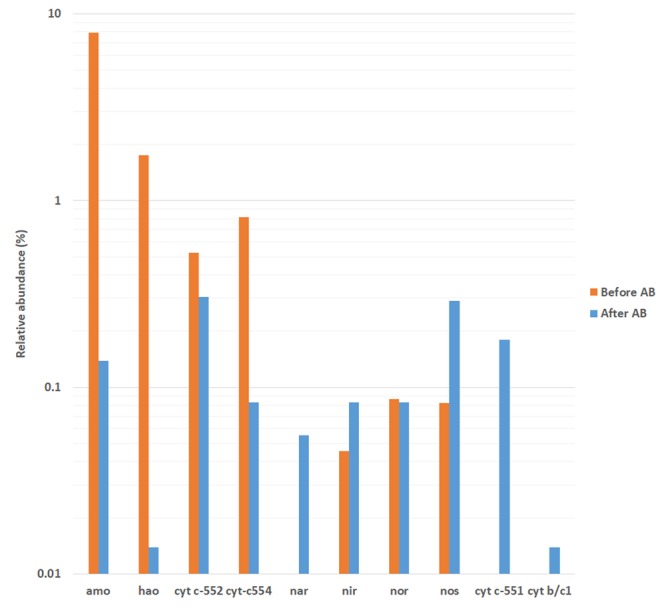
Relative abundance of genes implicated in autotrophic ammonium oxidation and heterotrophic nitrification-denitrification metabolisms as determined by metatranscriptomic analysis. Before AB: before the antibiotics addition; After AB: after the antibiotics addition.

The **Figure 6** highlights the activity involving the expression of proteins related to *amo* and *hao* genes, complex *cytochrome c-552* and *cytochrome c-554* genes for nitrification; and *nar*/*nap, nor, nir* and *nos* genes, *cytochrome b/c1* and *cytochrome c-551* for denitrification. As such, it was found that the antibiotics pressure drastically dropped the activity of ammonium oxidizing bacteria, observed in a decrease of ammonia monooxygenase and hydroxylamine oxidoreductase and the activity of complexes cytochrome c-552 and c-554 (with reductions of 98.25, 99.21, 41.84, and 89.83%, respectively). On the other hand, the addition of antibiotics caused an increase in the activities of nitrate reduction, nitrite reduction and nitrous oxide reduction. Moreover, the activity of complexes cytochromes b/c1 and c-551, which are related to heterotrophic nitrification-aerobic denitrification metabolisms (Guo et al., 2013), was only detected in operation under antibiotics. In this sense, as suggested by the determination of nitrogenous ions, the addition of antibiotics decreased the capacity of ammonium oxidation but increased the capacity of denitrification, which led to higher effluent ammonium and lower effluent total nitrogen concentrations, respectively. Also, as suggested by the analysis of bacterial community composition by 16S rRNA bacterial gene high-throughput sequencing, the antibiotics addition allowed the proliferation of bacteria with heterotrophic nitrification-aerobic denitrification capabilities, similarly to partial-nitritation biofilters subjected to ciprofloxacin (Gonzalez-Martinez et al., 2014b).

The results obtained showed that the addition of antibiotics to the partial-nitritation biofilter not only affected the microbial community structure, but also changed the metabolic pathways related to nitrogen. These results have been also reported by experiments in partial-nitritation biofilters subjected to ciprofloxacin antibiotic (Gonzalez-Martinez et al., 2014b). In this sense, the antibiotic compounds are potentially dangerous for partial-nitritation systems, causing severe losses in performance of ammonium oxidation to nitrite and therefore leading to system failure.

#### The Activity Related to Antibiotics Resistance

**FIGURE 7:**
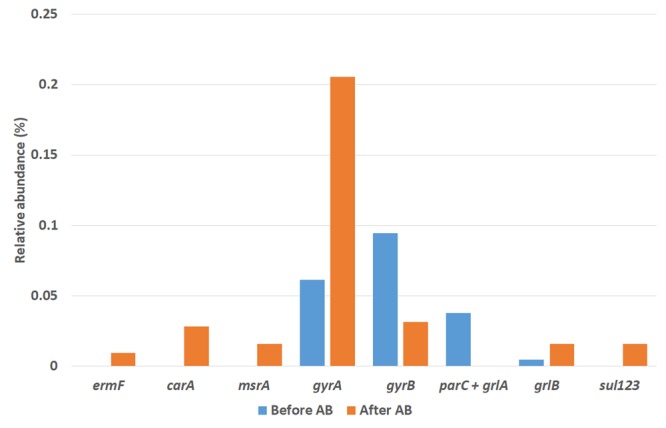
Relative abundance of antibiotics resistance genes found before and after the antibiotics addition as determined by metatranscriptomic analysis.

The presence of activities related to antibiotics resistance mechanisms was investigated within the mRNA identified. These activities were related to known resistance mechanisms to: macrolide-lincosamide-steptrogramin group (affiliated with genes *erm, car, msr, ole, smr, tlr, vga, vgb, lmr, sfa, mef, ere, lnu, vat* and *mph*), in which AZT is a member (Roberts, 2008); sulfonamides group (affiliated to genes *sul*), which counts SMZ (Wang et al., 2014); and fluoroquinolone group (affiliated with genes *gyr, par, grl, qnr, oqx, qep, pat, acr* and *tol*), in which NOR stands (Redgrave et al., 2014). Activities of these genes were only detected within the Bacteria domain. A graphical representation of the relative abundance of these genes before and after the addition of the antibiotics mix is given in **Figure 7**.

No macrolides-resistance genes were found before the antibiotics addition, but three were found at the end of the operation after antibiotics addition: target-site mutation ermF gene and efflux-related genes *carA* and *msrA*. Similarly to the AZT case, sulfonamide-resistance genes *sul1, sul2* and *sul3* were found only after the addition of the antibiotics. On the other hand, the only resistance genes against fluoroquinolone found after the antibiotics addition were *gyrA, gyrB*, and *grlB*. On the other hand, the activity of genes *parC*+*grlA* was only present after the antibiotics addition.

In this sense, it is possible that loading of antibiotics could trigger the emergence of resistance to these compounds within the bacterial community structure of the partial nitritation biofilter by the means of mutation leading to formation of dihydropteroate synthase with lower affinity for this antibiotic for sulfonamides; functioning of efflux-related genes against AZT; and by target-site mutation of DNA gyrase subunit A *gyrA* and DNA topoisomerase subunit B *grlB* for fluoroquinolone.

#### Activity Related to Carbon Metabolism

Metatranscriptomics data showed that there was a slight increase in pyruvate kinase enzyme, related to glycolysis, with the addition of antibiotics, which could indicate the preference of glycolysis when the antibiotics were present in the bioreactor (Supplementary Table S5). Also, enzymes alcohol dehydrogenase and lactate dehydrogenase, which are crucial for the fermentation of organic matter, were much higher after the antibiotics addition (2.5-fold increase in alcohol dehydrogenase after antibiotics additions, while no lactate dehydrogenase was found before the antibiotics loading), which seemed to indicate that fermentation of organic matter was more important after the addition of the antibiotics. The relative abundance of Krebs cycle enzymes was higher after the antibiotics addition than before. In this sense, it is possible that the loading of antibiotics caused an increase of the importance in the heterotrophic metabolism. Thus, the growth of the biofilm as the system adapted to the antibiotics loading could be related to the growth of heterotrophs. These microorganisms could grow by degradation of the antibiotics compound or by consumption of biofilm EPS and cell material. In this sense the loading of 377 mg day-1 of antibiotics could support the growth of heterotrophs. Since the removal of AZT coincided temporally with the near-maximum biofilm biomass, it is possible that AZT biodegradation could support the growth of the biofilm. Previous research on partial nitritation biofilters subjected to ciprofloxacin concluded that biofilm growth after antibiotics addition could be caused by ciprofloxacin degradation of dominant genus *Comamonas* (Gonzalez-Martinez et al., 2014b). The removal of high concentrations of norfloxacin, AZT and trimethoprim was related to an *Alcaligenes* strain (Rodriguez-Sanchez et al., 2017), which was taxonomically related to the dominant genus in the partial nitritation biofilter after the antibiotics addition. The data obtained in this research suggested that microbial community dynamics in bioreactors subjected to antibiotics could be related to heterotrophic metabolisms of antibiotics resistant bacteria, which could biodegrade the antibiotics. More research is necessary in order to fully unravel the mechanisms of biomass adaptation in bioreactors.

### Multivariate Redundancy Analysis Linking the Bacterial Community Structure with the Operational Conditions in the Partial-Nitritation Biofilter

**FIGURE 8:**
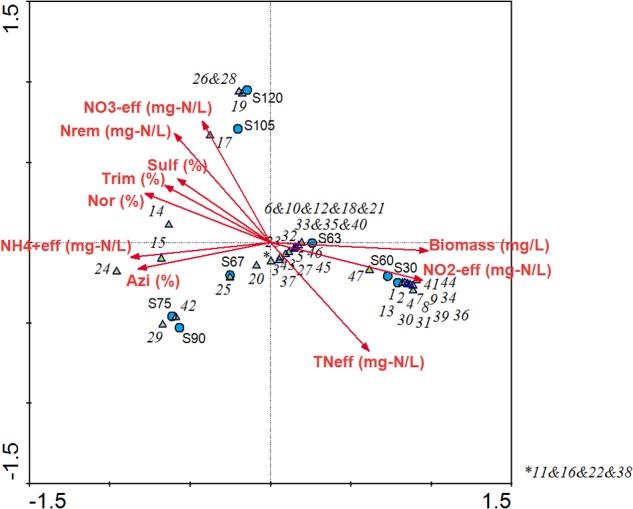
Multivariate redundancy analysis linking the performance of the partial-nitritation biofilter with the bacterial community structure during the operation time. The numbers from 1 to 47 are correlated with the microbial population codes in the **Figure 3**.

The multivariate redundancy analysis developed for the operation under antibiotics showed two groups of variables (**Figure 8**). Mainly, ammonium oxidation performance was negatively correlated with biomass concentration inside the system. In this sense, the observed loss of biomass after the antibiotics addition was strongly correlated with the loss in partial-nitritation performance. As shown by bacterial community dynamics, the loss of *Nitrosomonas* genus caused the decrease in effluent nitrite concentration after antibiotics loading to the biofilter. Genera *Alcaligenes* and *Acidovorax* showed a positive correlation with effluent nitrate concentration and nitrogen removal, which supports the nitrifying and denitrifying activities of both genera found by the metatranscriptomic analysis. *Brevundimonas, Paracoccus, Ochrobactrum*, and *Alcaligenes*, among others, were correlated with net antimicrobials elimination. These phylotypes were also found to be related to antibiotics removal in a CANON bioreactor subjected to an antibiotics mix of AZT, NOR, TMP, and SMZ (Rodriguez-Sanchez et al., 2017). These results showed that the same antibiotics-resistant genera proliferated in two different technologies with different biomass configurations and thus aim to the fact that the nature of the antibiotics compound was the most important driving factor for the bacterial communities in these bioreactors.

## Conclusion

A partial-nitritation biofilter was subjected to a high antibiotics concentration influent. The addition of antibiotics reduced the partial-nitritation efficiency. This was caused by the loss of biomass, as showed by multivariate redundancy analysis, and by changes in the bacterial community structure, as shown by high-throughput sequencing of 16S rRNA gene of *Bacteria* domain. The changes in the bacterial communities after the addition of the antibiotics suggested the proliferation of *Alcaligenes, Acidovorax*, and *Paracoccus*. Metatranscriptomics analysis demonstrated that dominant bacterial genera after the antibiotics addition expressed proteins that develop heterotrophic nitrification and aerobic denitrification metabolism, which contrasted with the high activity of ammonium oxidation found in the biofilter after the antibiotics addition. The addition of antibiotics could be related to an increase of aerobic and anaerobic heterotrophic metabolisms. The antibiotics caused a decrease in the bacterial activity within the system but an increase in the activities of *Archaea* and *Fungi* phylotypes. In this sense, the presence of the antimicrobial compounds also impacted small players in the performance of the partial-nitritation biofilter. Resistance to AZT, sulfonamides and norfloxacin were related to *ermF, carA, msrA, sul123, gyrA*, and *grlB* genes. The coupled analysis using bioreactor’s performance, bacterial community structure and metatranscriptomics analysis offered a complete comprehensive understanding of the influence of the antibiotics in the bioreactor analyzed during the experiment. The results showed that the addition of the antibiotics reduced the ammonium oxidation efficiency of the system and enhanced the nitrogen removal capacity, which was correlated with the decrease of autotrophic ammonium oxidizing bacteria, the proliferation of heterotrophs a decrease in ammonium oxidation activity and an increase in the denitrification activity inside the bioreactor. The results linked the performance of the bioreactor, the bacterial community dynamics and the microbial activity in the system in order to reach a complete approach for the evaluation of the antibiotics effect over the system, and will be valuable for the treatment of effluents with high antibiotics concentrations.

## Author Contributions

AG-M is the main researcher in the bioreactor, physico-chemical and molecular biology techniques. AM is the main researcher in micropollutants studies. AR-S has worked on bioinformatic analysis. CP has worked on molecular biology techniques. SD-C supervised the micropollutant studies and the manuscript preparation. DB has supervised the micropollutant studies and the whole research results. RV has supervised the engineering, molecular biology results, and the whole research results.

## Conflict of Interest Statement

The authors declare that the research was conducted in the absence of any commercial or financial relationships that could be construed as a potential conflict of interest.

## Footnotes

1 http://rnacentral.org/

2 ftp://ftp.ncbi.nlm.nih.gov/refseq/release/

3 ftp://ftp.ncbi.nlm.nih.gov/blast/db/
