# Supplementary material for: Linking the Effect of Antibiotics on Partial-Nitritation Biofilters: Performance, Microbial Communities and Microbial Activities
Source: Front Microbiol. 2018 Feb 26;9:354. doi: 10.3389/fmicb.2018.00354 (PMC5834488; doi:10.3389/fmicb.2018.00354)
Supplement: Supplementary file 1 [file Table_1.DOCX]

**Performance, metagenomic and metatranscriptomic studies in a submerged-bed partial nitritation bioreactor under high antibiotics concentrations**

Alejandro Gonzalez-Martinez^a^, Alejandro Margareto^b,c^, Alejandro Rodriguez-Sanchez^d^, Ana Martin-Garcia^d^, Chiara Pesciaroli^d^, Silvia Diaz-Cruz^b,c^, Damia Barcelo^b,c^, Riku Vahala^a^,

^a^: Department of Built Environment, School of engineering, Aalto University, P.O. Box 15200, Aalto, FI-00076 Espoo, Finland.

^b^: Department of Environmental Chemistry, Institute of Environmental Assessment and Water Research. Spanish Council for Scientific Research (IDAEA-CSIC), Jordi Girona 18-26, 08034 Barcelona, Spain

^c^: Catalan Institute for Water Research (ICRA), Scientific and Technological Park of the University of Girona, H2O Building, Emili Grahit 101, E17003 Girona, Spain

^d^: Institute of Water Research, University of Granada, Ramón y Cajal, 4, 18071 Granada, Spain.

* Corresponding author: Alejandro Gonzalez-Martinez, Department of Built Environment, Aalto University, P.O. Box 15200, Aalto, FI-00076 Espoo, Finland; E-mail: [alejandro.gonzalezmartinez@aalto.fi](mailto:alejandro.gonzalezmartinez@aalto.fi)

**Supplementary material**

Table S1 – MS/MS optimized experimental parameters and transitions monitored for the target antibiotics. In all cases the precursor ion was [M+H]^+^.

Table S2 – Performance of the SPE-HPLC-MS/MS method applied for the analysis of antibiotics.

*Average recovery value from the 3 concentration levels of recovery experiments.

Table S3 – Coverage analysis of the massive parallel sequencing subsamples

| Sample |  | Reads | Species richness | Good's coverage |  | Coverage (%) | Actual effort (Mbp) | Required effort (Mbp) |
| --- | --- | --- | --- | --- | --- | --- | --- | --- |
| S30 |  | 15778 | 377 | 0.97611 |  | 95.13 | 19.56 | 1.535 |
| S60 |  | 15778 | 405 | 0.97433 |  | 95.88 | 18.13 | 1.547 |
| S63 |  | 15778 | 309 | 0.98042 |  | 94.81 | 2.752 | 0.7286 |
| S67 |  | 15778 | 251 | 0.98409 |  | 93.79 | 2.033 | 0.72 |
| S75 |  | 15778 | 183 | 0.98840 |  | 94.21 | 1.424 | 0.4699 |
| S90 |  | 15778 | 122 | 0.99227 |  | 92.5 | 1.028 | 0.2549 |
| S105 |  | 15778 | 104 | 0.99341 |  | 94.07 | 0.9128 | 0.1203 |
| S120 |  | 15778 | 113 | 0.99284 |  | 95.3 | 2.186 | 0.1159 |

Table S4 – α-diversity of massive parallel sequencing subsamples

| Samples | Chao-1 | Shannon-Wiener | Simpson | Pielou's evenness | Berger-Parker |
| --- | --- | --- | --- | --- | --- |
| S30 | 552.1 | 2.889 | 0.8008 | 0.4869 | 0.42350 |
| S60 | 596.6 | 3.141 | 0.8475 | 0.5232 | 0.34170 |
| S63 | 402.4 | 3.884 | 0.9649 | 0.6774 | 0.08506 |
| S67 | 353.1 | 2.682 | 0.8129 | 0.4855 | 0.38690 |
| S75 | 294.1 | 1.753 | 0.6374 | 0.3365 | 0.55860 |
| S90 | 200.4 | 1.496 | 0.6456 | 0.3113 | 0.46150 |
| S105 | 149.9 | 1.833 | 0.7821 | 0.3947 | 0.29430 |
| S120 | 162.3 | 1.736 | 0.7470 | 0.3671 | 0.36300 |

Table S5 – Relative abundance of activities related to carbon metabolisms before and after the antibiotics addition

| Activity | Before | After |
| --- | --- | --- |
| Pyruvate kinase | 0.02832727 | 0.03163556 |
| Lactate dehydrogenase | 0 | 0.01581778 |
| Alcohol dehydrogenase | 0.01888485 | 0.04745334 |
| Combined Krebs Cycle | 0.27855153 | 0.36380892 |


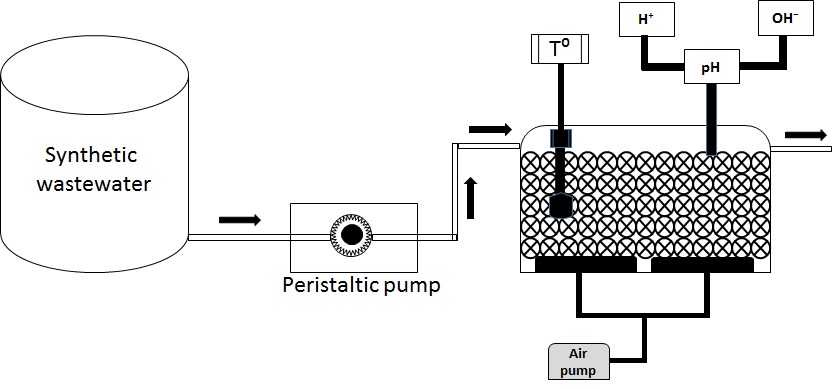


Figure S1 – Scheme of the partial-nitritation biofilter


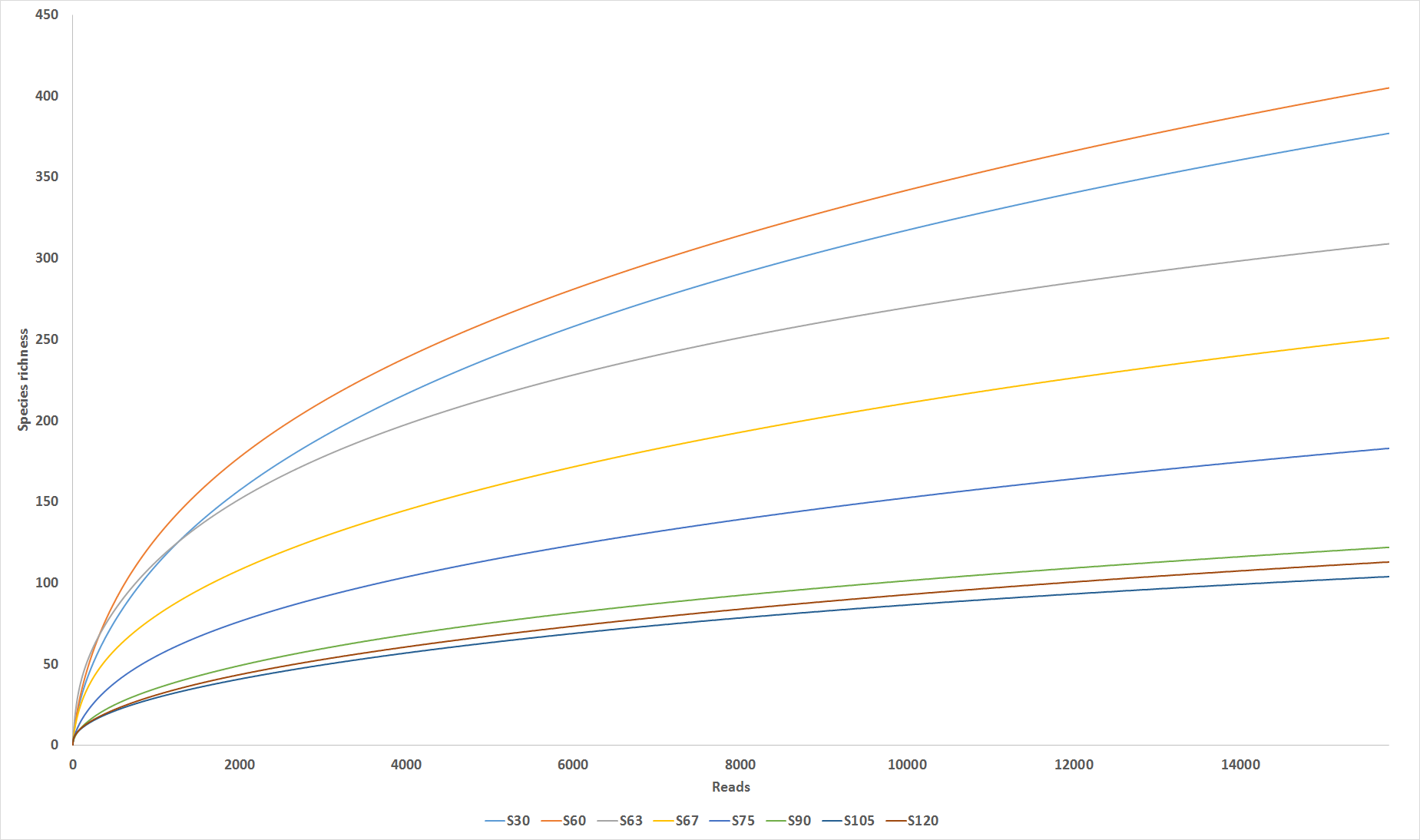


Figure S2 – Complexity curves of the massive parallel sequencing subsamples


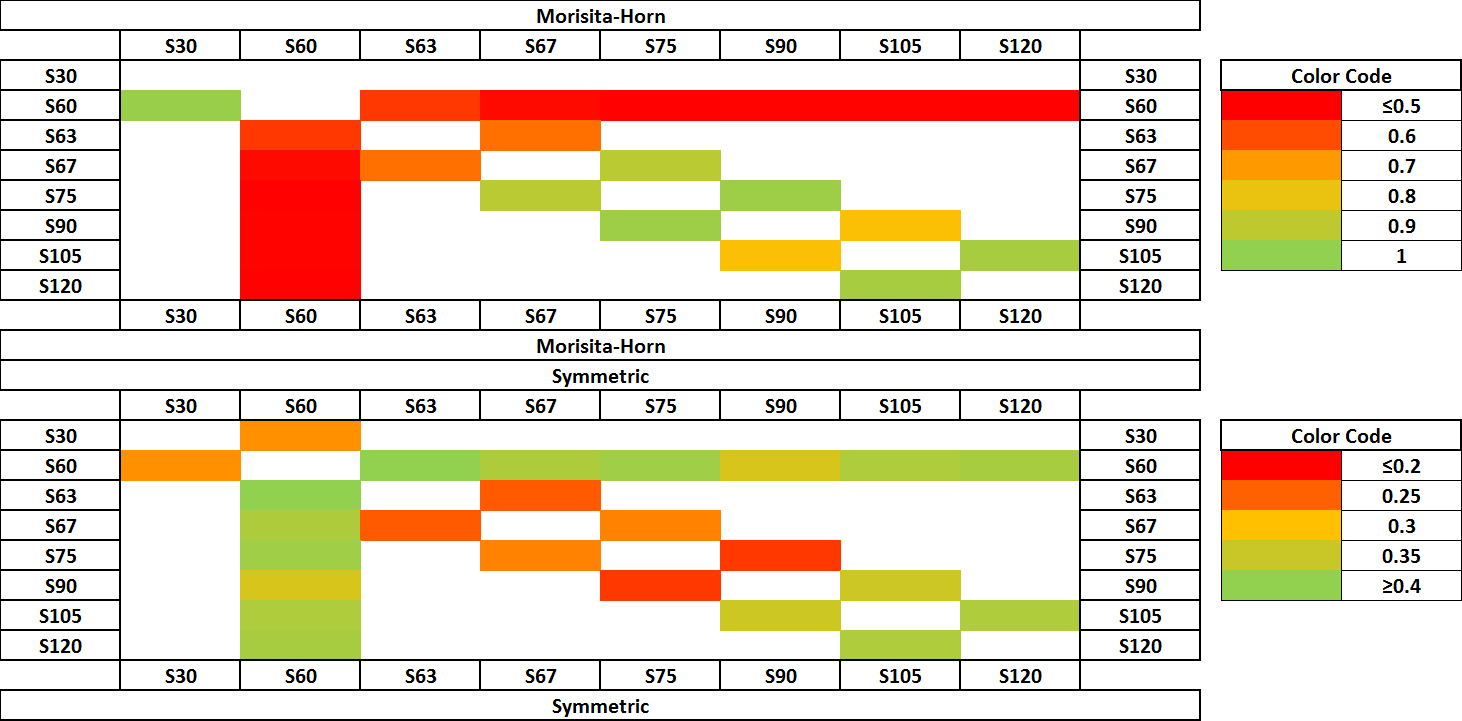


Figure S3 – β-diversity analysis of the massive parallel sequencing subsamples
